# Adipocyte-derived exosomal miR-22-3p modulated by circadian rhythm disruption regulates insulin sensitivity in skeletal muscle cells

**DOI:** 10.1016/j.jbc.2023.105476

**Published:** 2023-11-18

**Authors:** Haohao Zhang, Xiaoning Zhang, Saifei Wang, Lu Zheng, Hengru Guo, Yanqi Ren, Bo Qiao, Jing Wu, Di Zhao, Lijun Xu, Shengnan Ma, Xiao Hao, Yushan Yan

**Affiliations:** 1 Department of Endocrinology, The First Affiliated Hospital of Zhengzhou University, Zhengzhou, China; 2 Department of Endocrinology, The Third People's Hospital of Zhengzhou, Zhengzhou, China; 3 Department of Endocrinology, Changzhi Medical College Affiliated Heping Hospital, Changzhi, China; 4 Department of Pediatrics, The First Affiliated Hospital of Zhengzhou University, Zhengzhou, China

**Keywords:** circadian rhythm disruption, exosomes, miR-22-3p, insulin resistance, adipocytes, skeletal muscle

## Abstract

Circadian rhythm disruption leads to dysregulation of lipid metabolism, which further drive the occurrence of insulin resistance (IR). Exosomes are natural carrier systems that advantageous for cell communication. In the present study, we aimed to explore whether and how the exosomal microRNAs (miRNAs) in circulation participate in modulating skeletal muscle IR induced by circadian rhythm disruption. In the present study, 24-h constant light (12-h light/12-h light, LL) was used to establish the mouse model of circadian rhythm disruption. Bmal1 interference was used to establish the cell model of circadian rhythm disruption. And in clinical experiments, we chose a relatively large group of rhythm disturbance-shift nurses. We showed that LL-induced circadian rhythm disruption led to increased body weight and visceral fat volume, as well as occurrence of IR *in vivo*. Furthermore, exosomal miR-22-3p derived from adipocytes in the context of circadian rhythm disruption induced by Bmal1 interference could be uptaken by skeletal muscle cells to promote IR occurrence *in vitro*. Moreover, miR-22-3p in circulation was positively correlated with the clinical IR-associated factors. Collectively, these data showed that exosomal miR-22-3p in circulation may act as potential biomarker and therapeutic target for skeletal muscle IR, contributing to the prevention of diabetes in the context of rhythm disturbance.

In mammals, many behavioural and physiologic processes including the sleep–wake cycle, feeding behaviour and energy metabolism, exhibit 24-h rhythms. Such 24-h rhythms (‘about a day’) are temporally coordinated by the endogenous circadian clocks and hence the body can keep self-sustained rhythms by exposure to the light-dark cycle (1). However, night-shift work or exposure to artificial light at night could led to the disruption of circadian rhythm (2, 3).

Multiple lines of evidence indicate circadian rhythm disruption leads to dysregulation of lipid metabolism and obesity, which further drive the occurrence of insulin resistance (IR) (4, 5, 6). IR is a crucial pathophysiological process in the development of type 2 diabetes mellitus (T2DM) and a main cause of morbidity and mortality (7). It has been reported that T2DM begins with the progression of peripheral IR, which usually originates within the skeletal muscle (8). Although a growing body of research have proposed numerous mechanisms for the development of skeletal muscle IR, such as classical glucose handling, mitochondrial degeneration, and dysregulation of microRNAs (miRNAs) (9), how the disruption of circadian rhythm induced skeletal muscle IR remains preliminary. Improving understanding of the regulatory mechanism underlying skeletal muscle IR in the context of circadian rhythm disruption may be therefore pivotal for developing effective therapeutic targets to prevent the progression of T2DM.

Exosomes are membrane-bound extracellular vesicles that are generated from multivesicular bodies of most mammalian cell types, and then are released into the extracellular space through fuse with the plasma membrane (10, 11). MiRNAs are common cargoes that can be loaded into exosomes. MiRNAs are small non-coding RNAs (about 22 nucleotides long) that participate in a series of physiological and pathological processes by negatively regulating gene expression (12). As effective carriers, exosomes either transfer these miRNAs to the neighboring cells, or enter the circulation to transport miRNAs to the distant cells, thereby regulating the function of the recipient cells.

In the present study, we found that the circulating exosomal miR-22-3p was upregulated in the mouse model of circadian rhythm disruption induced by constant light through analyzing the miRNA profile of the plasma exosomes, suggesting that exosomal miR-22-3p plays an important role in modulating the functions of circadian rhythm disruption. It has been reported that miR-22-3p inhibition could lead to a potent treatment of IR and related metabolic disorders like obesity (13, 14), indicating that miR-22-3p up-regulation may participate in the skeletal muscle IR. However, the origin and exact role of such upregulated exosomal miR-22-3p in circulation need to be clarified. In the present study, we explored the source and the function of circulating exosomal miR-22-3p in the context of circadian rhythm disturbance.

## Results

### Constant light induced circadian rhythm disruption and IR *in vivo*

**Figure 1:**
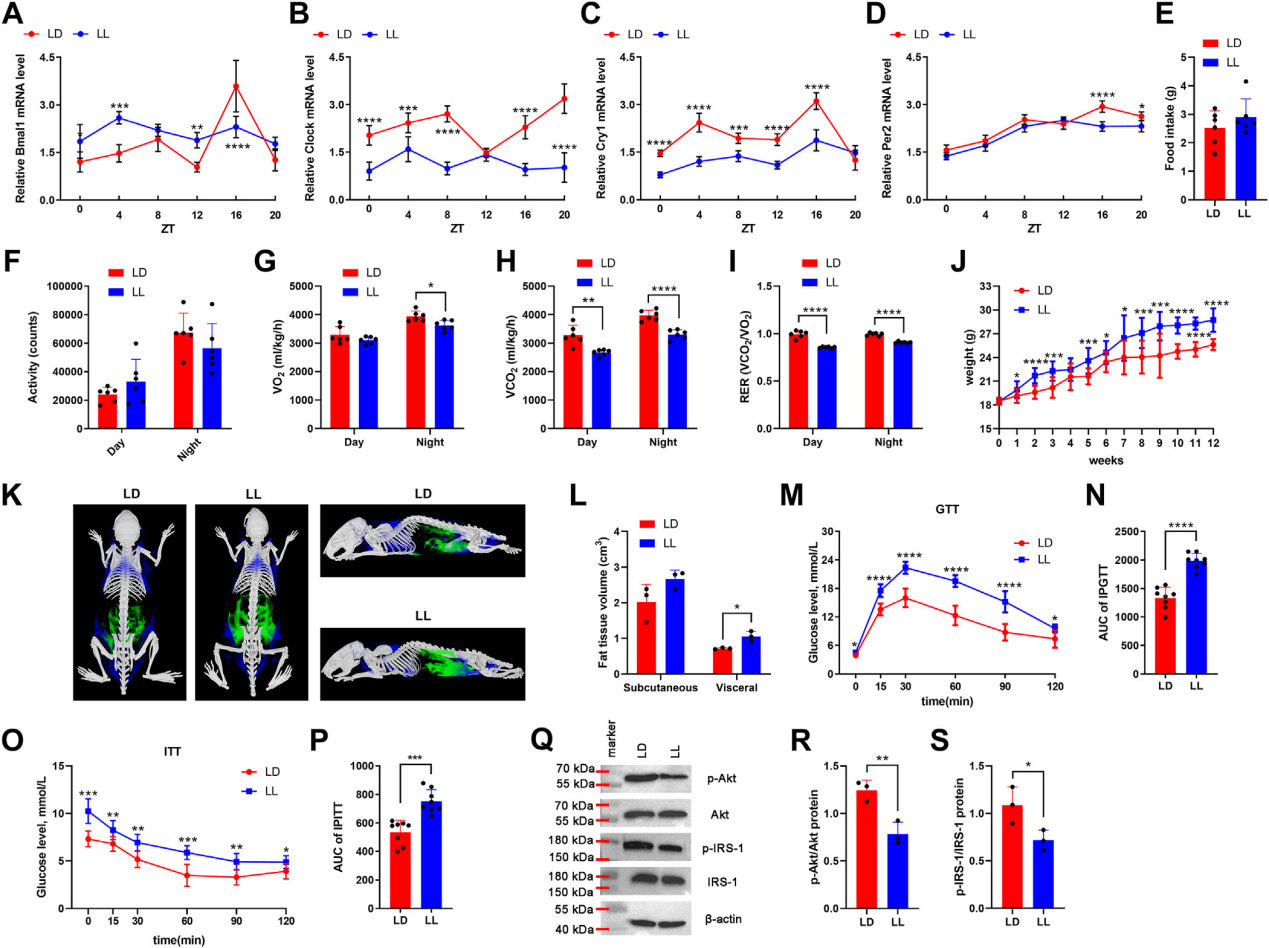
**Constant light-induced circadian rhythm disruption and IR *in vivo*.** The mRNA expression of Bmal1 (*A*), Clock (*B*), Cry1 (*C*), and Per2 (*D*) in monocytes from peripheral blood collected at different ZTs (ZT0-ZT20) was detected by qPCR assay. The food intake (*E*), activity (*F*), VO_2_ (*G*), and VCO_2_ (*H*) levels were assessed by CLAMS assay. *I*, RER was calculated by VCO_2_/VO_2_. *J*, the weight of the mice was monitored once a week for 12 weeks. *K* and *L*, the subcutaneous and visceral fat content were measured and analyzed by Micro-CT assay (*Blue*: subcutaneous fat; *Green*: visceral fat). The GTT (*M*) and ITT (*O*) were performed by using a glucometer. The AUC of GTT (*N*) and ITT (*P*) were calculated. *Q*, the expression of insulin signaling pathway-related protein p-Akt, Akt, p-IRS-1, and IRS-1, in skeletal muscle tissues were detected by Western blot assay. The p-Akt/Akt (*R*) and p-IRS-1/IRS-1 (*S*) protein ratio were shown. ∗*p* < 0.05; ∗∗*p* < 0.01; ∗∗∗*p* < 0.001; ∗∗∗∗*p* < 0.0001.

**Fig. S1:**
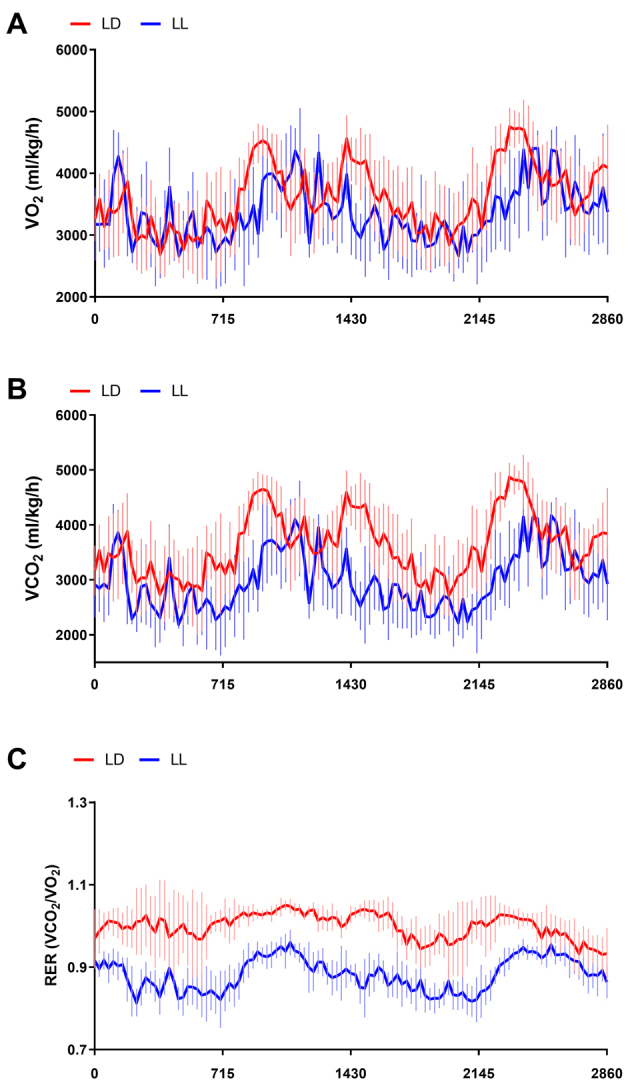
**Constant light-induced disordered energy metabolism.** VO_2_ (*A*), VCO_2_ (*B*), and RER (*C*) levels were assessed by CLAMS assay.

We established a mouse model of circadian rhythm disruption with 10 weeks of 24-h constant light (12-h light/12-h light, LL), characterized by the disordered rhythm of Bmal1, Clock, Cry1 and Per2 mRNA expression in monocytes from peripheral blood (Fig. 1, *A*–*D*). Compared with the 12-h light/12-h dark cycle (LD) group, the food intake and daytime activity showed an increase, while night activity showed a decrease trend in the LL group, but the differences were not significant (Fig. 1, *E* and *F*). The CLAMS assay showed that compared with the LD group, the VO_2_ level at night were significantly decreased in the LL group, although there were no markedly differences in daytime VO_2_ levels between the two groups; However, VCO_2_ and RER levels in the LL group were significantly decreased compared with those in the LD group, both during the day and at night (Figs. 1, *G*–*I* and S1). Furthermore, compared with LD group, the body weight of LL group was significantly higher than that of control group from the second week, and the body weight showed a 11% increase after 12 weeks of continuous illumination (Fig. 1*J*). The Micro-CT assay showed the subcutaneous and visceral fat volume were both increased after 12 weeks of continuous illumination, although only the visceral fat volume showed significantly difference (Fig. 1, *K* and *L*). Moreover, GTT results showed that the blood glucose level of both groups reached the peak value at 30 min, but the level and increase amplitude of blood glucose in LL group were significantly higher than that in LD group (Fig. 1*M*). ITT results showed that the blood glucose level in LD group reached the lowest value at 60 min, while the blood glucose level in LL group decreased slowly and reached the lowest value at 90 min (Fig. 1*O*). The area under the curve (AUC) of GTT (Fig. 1*N*) and ITT (Fig. 1*P*) in LL group were both significantly higher than those in LD group. Western blot assay showed that the p-IRS-1/IRS-1 and p-Akt/Akt level were both decreased in LL group compared with LD group (Fig. 1, *Q*–*S*).

### Constant light induced upregulated exosomal miR-22-3p in circulation *in vivo*

**Figure 2:**
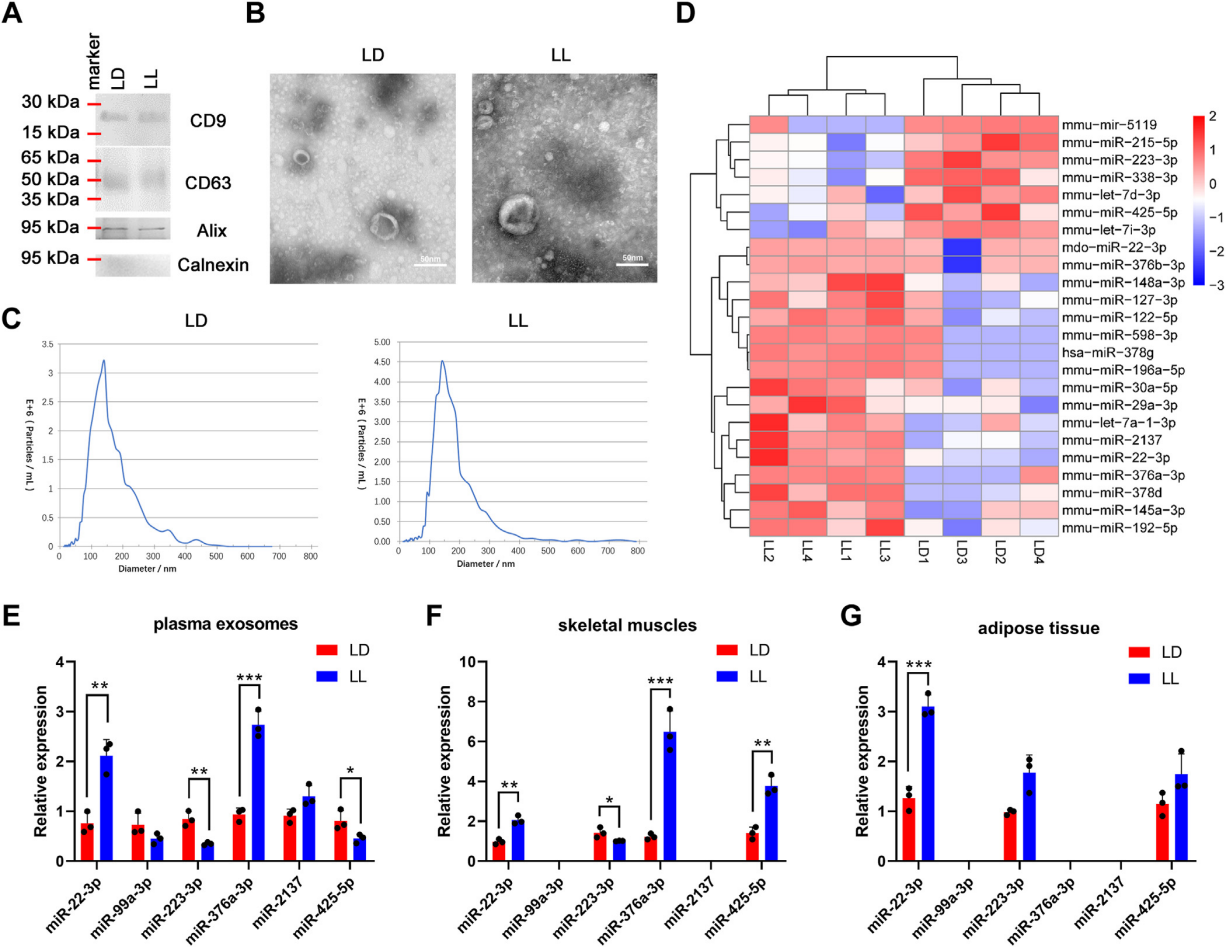
**Constant light-induced upregulated exosomal miR-22-3p in circulation *in vivo*.***A*, the protein expression of CD9, CD63, Alix, and Calnexin in plasma exosomes were detected by Western blot assay. The plasma exosomes were analyzed by (*B*) transmission electron microscopy and (*C*) NTA assay. *D*, the differentially expressed miRNAs in the plasma exosomes were shown in bicluster analysis. The expression of miR-22-3p, miR-99a-3p, miR-223-3p, miR-376a-3p, miR-2137, and miR-425-5p in plasma exosomes (*E*), skeletal muscle tissues (*F*), and visceral adipose tissues (*G*) were measured by qPCR, respectively. ∗*p* < 0.05; ∗∗*p* < 0.01; ∗∗∗*p* < 0.001.

We have showed that the circadian rhythm disruption induced by LL treatment could further lead to IR. However, the underlying regulatory mechanism need more investigation. We further extracted the plasma exosomes from LD group and LL group, respectively, which was evidenced by the expression of CD9, CD63 (transmembrane protein that prove the existence of lipid bilayer) and Alix (cytoplasmic protein that proves the extracellular vesicles contain more cytoplasmic material than cell fragments), as well as the absence of Calnexin (to confirm the absence of contamination from other cellular components, Fig. 2*A*). In addition, transmission electron microscopy showed obvious cup-shaped membranous vesicles with diameters ranging from 50 nm to 120 nm (Fig. 2*B*). NTA assay showed that particle size distribution of both the two groups ranged from 50 nm to 200 nm, with an average particle size of about 140 nm (Fig. 2*C*). Then we conducted high-throughput sequencing to perform differential expression profile of the exosomal miRNAs, and found that compared with the LD group, 24 exosomal miRNAs exhibited significantly changed in the LL group. Among them, 17 miRNAs were significantly up-regulated and seven were significantly down-regulated. Further bicluster analysis of the differentially expressed miRNAs showed that the plasma exosomes secreted by both the two groups had good repeatability and obvious classification (Fig. 2*D*). Among the differentially expressed miRNAs, miR-22-3p, miR-99a-3p, miR-223-3p, miR-376a-3p, miR-2137 and miR-425-5p reportedly associates with energy metabolism (15, 16, 17, 18, 19). Thus we performed qPCR to verify their expression in plasma exosomes, skeletal muscle tissues, and visceral adipose tissues, respectively. As shown in Figure 2, *E*–*G*, miR-22-3p showed significantly upregulated in all the three parts, implying a button molecule that connects the dialogue between adipose tissues and skeletal muscle tissues.

### Elevated expression of miR-22-3p in circulation positively correlated with IR-relative factors clinically in the context of circadian rhythm disruption

**Figure 3:**
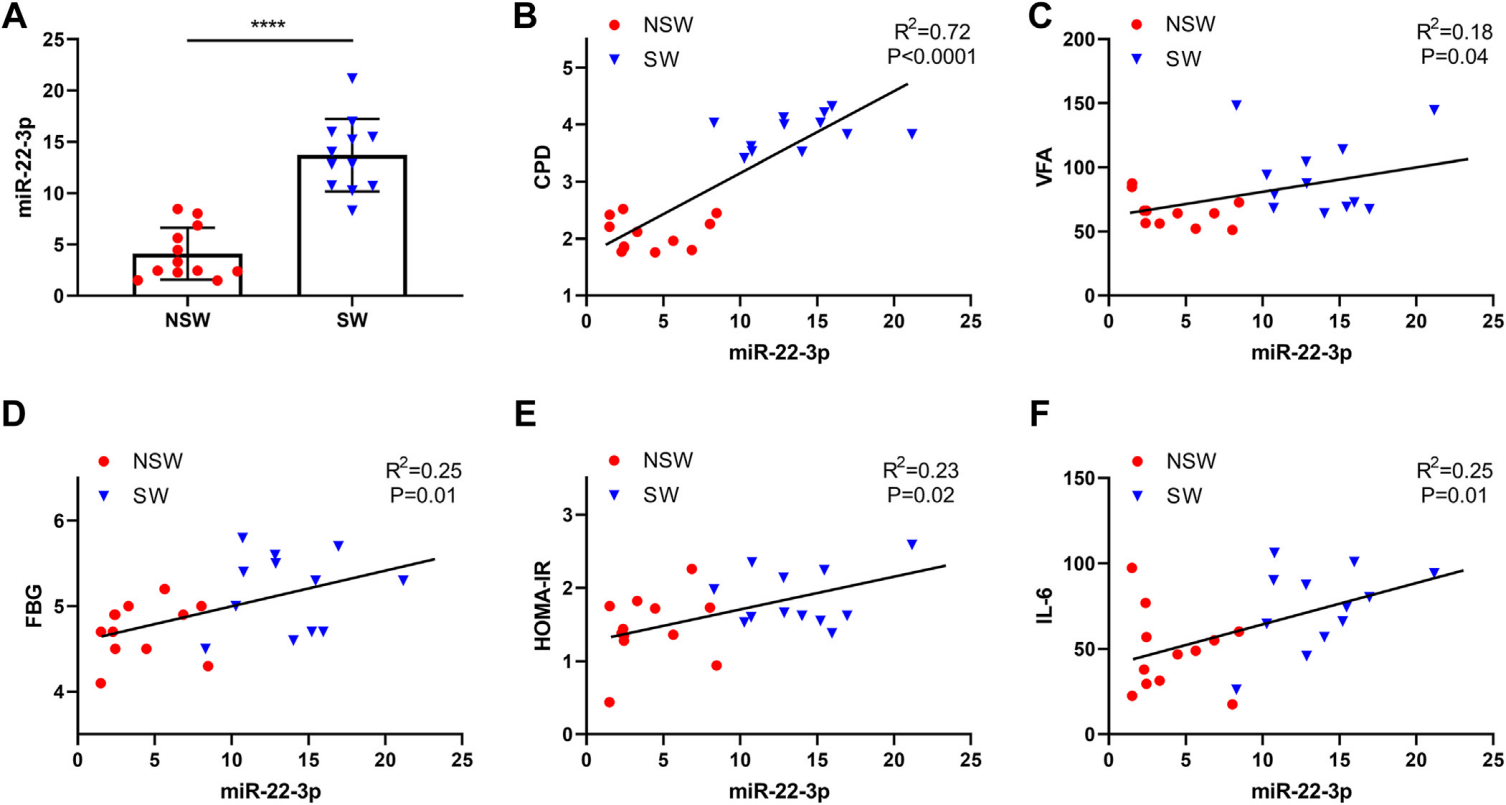
**MiR-22-3p expression in circulation positively correlated with IR-relative factors clinically.** Twelve shift nurses (shift working group, SW group) and twelve non-shift nurses (non-shift working group, NSW group) were enrolled. *A*, the expression of miR-22-3p in plasma was assessed by qPCR assay. ∗∗∗∗*p* < 0.0001. The mid-sleep time compound phase deviation (CPD) was calculated based on a questionnaire. Visceral fat area (VFA) was evaluated by bioelectrical impedance analysis. The fasting blood glucose (FBG) was detected by glucose dehydrogenase assay. The fasting insulin was detected by chemiluminescence particle immunoassay. Homeostasis model assessment of IR (HOMA-IR) = fasting blood glucose (mmol/L) × fasting insulin (μIU/ml)/22.5. Serum IL-6 levels were detected by ELISA assay. The correlation between miR-22-3p expression and CPD (*B*), VFA level (*C*), FBG level (*D*), HOMA-IR (*E*), and IL-6 level (*F*) were performed by Spearman correlation analysis, respectively.

We further confirmed that the differential expression of miR-22-3p between shift working (SW) nurses and non-shift working (NSW) nurses. As shown in Figure 3*A*, miR-22-3p expression in the SW group showed significantly elevated compared with that in the NSW group. The composite phase deviation (CPD) and the IR-relative factors including VFA, fasting blood glucose (FBG), HOMA-IR, and IL-6, were further evaluated, and they were all obviously increased in the SW group compared with those in the NSW group (Table S3), indicating that shift work affected the stability of circadian clock in women, and could increase visceral fat and decrease insulin sensitivity before being overweight/obese. Furthermore, we conducted the correlation analysis between miR-22-3p expression and the above IR-relative factors, and the results showed that the expression of miR-22-3p in circulation positively correlated with each of the above factors (Fig. 3, *B*–*F*).

### MiR-22-3p was involved in the regulation of skeletal muscle IR *in vitro*

**Figure 4:**
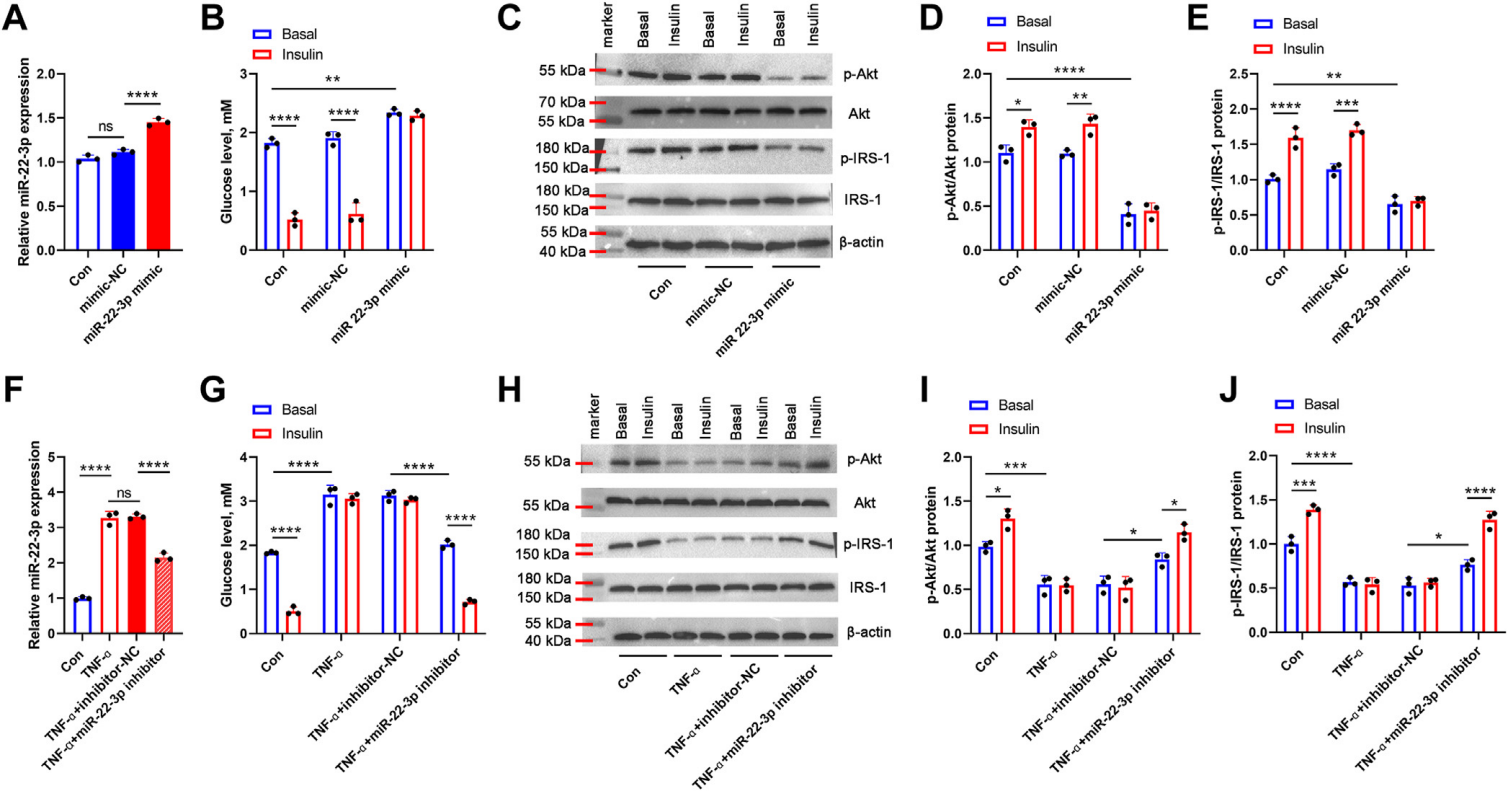
**MiR-22-3pwas involved in the regulation of skeletal muscle IR *in vitro*.** The C2C12 cells were randomly divided into con group, mimic-NC group, and miR-22-3p mimic group. Meanwhile, a cell model of IR was constructed by using TNF-α induction, and the C2C12 cells were randomly divided into con group, TNF-α group, TNF-α + inhibitor-NC group, and TNF-α+miR-22-3p inhibitor group. *A* and *F*, the expression of miR-22-3p was measured by qPCR. *B* and *G*, the glucose levels in the absence or presence of insulin were detected by using a glucose assay kit. *C* and *H*, the protein levels of p-Akt, Akt, p-IRS-1, IRS-1 were measured by Western blot assay. The p-Akt/Akt (*D* and *I*) and p-IRS-1/IRS-1 (*E* and *J*) protein ratio were shown.∗*p* < 0.05; ∗∗*p* < 0.01; ∗∗∗*p* < 0.001; ∗∗∗∗*p* < 0.0001.

We further explore the role of miR-22-3p in the IR of C2C12 cells. MiR-22-3p mimic transfection significantly elevated miR-22-3p expression in the C2C12 cells compared with mimic-NC transfection (Fig. 4*A*). As shown in Figure 4*B*, in the con group or the mimic-NC group, the glucose levels in the supernatant were significantly decreased after adding insulin, suggesting the insulin sensitivity in both the two group; the glucose levels in the supernatant had no significant changes in the absence or presence of insulin when miR-22-3p was overexpressed, which suppressed the glucose uptake, suggesting miR-22-3p overexpression contributed to IR occurence in C2C12 cells. Moreover, the protein levels of p-Akt and p-IRS-1 was significantly increased in the con or mimic-NC group after adding insulin, while the protein levels of p-Akt and p-IRS-1 showed no obvious differences in the miR-22-3p mimic group in the absence or presence of insulin, indicating that miR-22-3p overexpression contributed to IR occurrence by regulating IR-related protein levels (Fig. 4, *C*–*E*). Furthermore, we constructed a cell model of IR using TNF-α induction, accompanied by upregulated miR-22-3p expression (Fig. 4*F*). We speculated that inhibition of miR-22-3p could suppress TNF-α-induced IR in C2C12 cells. As indicated in Figure 4, *G*–*J*, compared with the TNF-α + inhibitor-NC group, miR-22-3p downregulation decreased glucose levels in the supernatant, and upregulated p-Akt/Akt and p-IRS-1/IRS-1 protein ratio in the TNF-α-induced IR cells, and the effects were more obvious after adding insulin, suggesting that miR-22-3p downregulation could elevate the insulin sensitivity in IR cells.

### The upregulated exosomal miR-22-3p in circulation was derived from adipocytes in the context of circadian rhythm disruption

**Figure 5:**
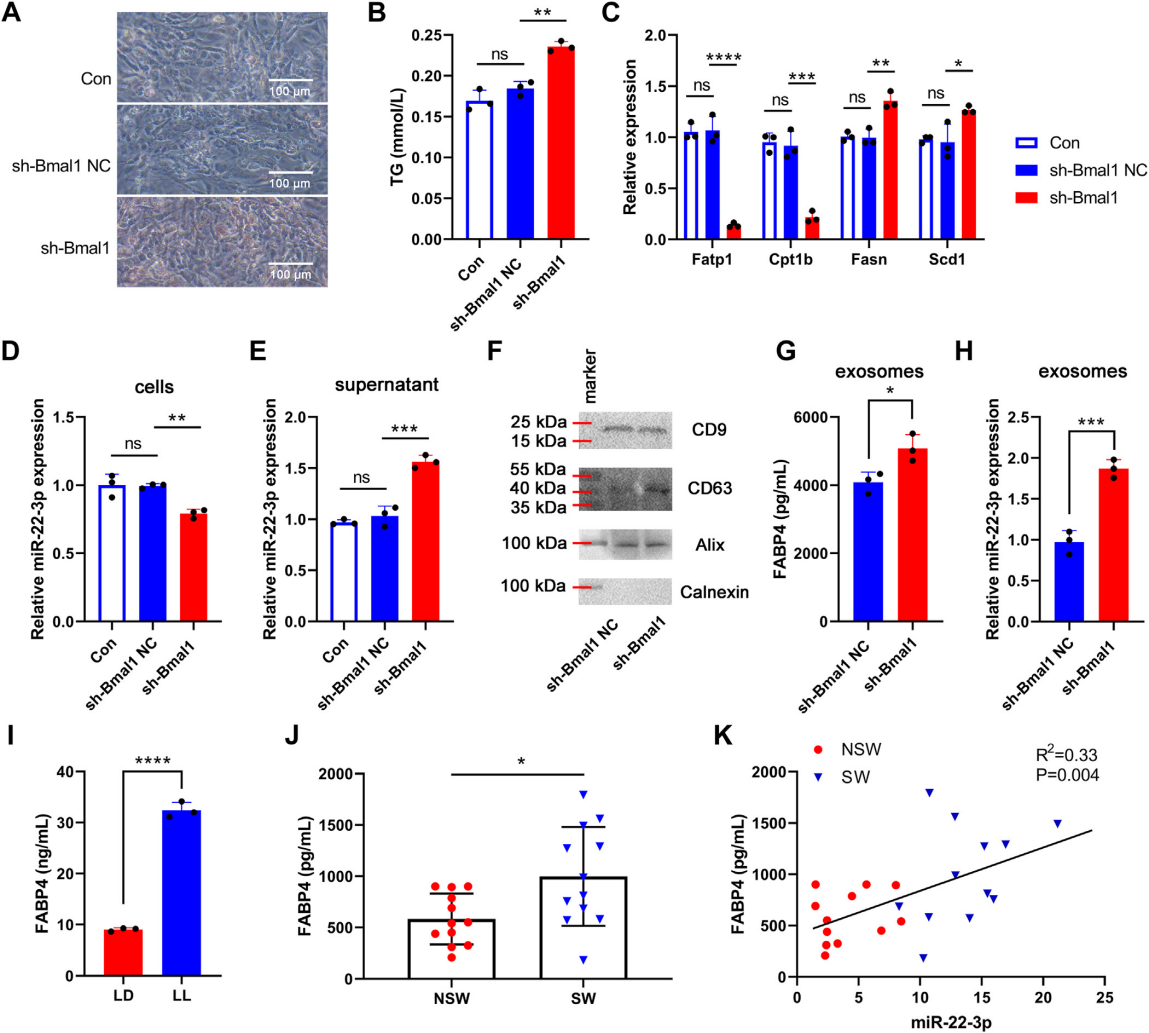
**The upregulated exosomal miR-22-3p in circulation was derived from adipocytes in the context of circadian rhythm disruption.***A*, oil red-O staining was performed to assess the adipogenesis. Scale bar represents 100 μm. *B*, intracellular TG content was determined by glycerol 3-phosphate oxidase method. *C*, the mRNA expression of fatty acid oxidase (Fatp1, Cpt1b) and fatty acid synthetase (Fasn, Scd1) were detected by qPCR. The expression of miR-22-3p in 3T3-L1 adipocytes (*D*) and in the supernatants (*E*) was measured by qPCR, respectively. *F*, the protein expression of CD9, CD63, Alix, and Calnexin in exosomes was detected by Western blot assay. *G*, the level of FABP4 in exosomes was assessed by ELISA. *H*, the expression of miR-22-3p in exosomes was measured by qPCR. The levels of FABP4 in serum form mouse model (*I*) and nurse samples (*J*) were assessed by ELISA, respectively. *K*, the correlation between miR-22-3p expression and FABP4 level in nurse samples was analyzed with Spearman correlation analysis. ∗*p* < 0.05; ∗∗*p* < 0.01; ∗∗∗*p* < 0.001; ∗∗∗∗*p* < 0.0001.

We had shown that miR-22-3p, which was upregulated in circulating exosomes in circadian rhythm disruption mouse model, was positively correlates with IR-relative factors clinically and was involved in the regulation of skeletal muscle IR *in vitro*. Thus, we further investigated the source of circulating exosomes with high expression of miR-22-3p, and explored whether these exosomes could act on skeletal muscle and participate in skeletal muscle IR. Studies have shown that adipose tissue is the main source of circulating exosomal miRNAs in obesity (20, 21), suggesting that circulating exosomal miR-22-3p in circadian rhythm disruption-induced obesity mice may be derived from adipose tissue. Basic helix-loop-helix ARNT like 1 (Bmal1), as a circadian gene, is a key regulator of adipogenesis, and inhibition of Bmal1could lead to adipogenesis (22). Thus the 3T3-Ll adipocytes were transfected with sh-Bmal1 to mimic the accumulation of lipid drop in adipocytes in the context of circadian rhythm disruption. As shown in Figure 5*A*, compared with the sh-Bmal1 NC group, oil red O staining confirmed increased lipid staining in 3T3-L1 adipocytes transfected with sh-Bmal1, indicative of enhanced adipogenesis. Consistent with this, the TG level in the sh-Bmal1 group was significantly elevated compared with that in the sh-Bmal1 group (Fig. 5*B*). Meanwhile, the expression of adipogenesis-associated genes were measured. As shown in Figure 5*C*, compared with the sh-Bmal1 NC group, the mRNA expression of fatty acid oxidase (Fatp1, Cpt1b) was significantly suppressed, while the mRNA expression of fatty acid synthetase (Fasn, Scd1) was markedly increased in the sh-Bmal1 group.

We further explored the effect of adipogenesis by Bmal1 interference on exosome release in 3T3-L1 adipocytes. The miR-22-3p expression was decreased in the 3T3-L1 adipocytes (Fig. 5*D*), but was elevated in the supernatant (Fig. 5*E*), indicating that miR-22-3p may be released more from 3T3-L1 adipocytes through exosomes in the context of Bmal1 interference-induced adipogenesis. Furthermore, the exosomes were extracted from the sh-Bmal1 NC group and sh-Bmal1 group, respectively, which was evidenced by the expression of CD9, CD63 and Alix, as well as the absence of Calnexin (Fig. 5*F*). Compared with the sh-Bmal1 NC group, the expression of fatty acid binding protein 4 (FABP4, a specific marker of exosome derived from adipocytes) and miR-22-3p in the exosomes from the sh-Bmal1 group were both up-regulated compared with those in the exosomes from the sh-Bmal1 NC group (Fig. 5, *G* and *H*). These data validated that adipocytes interfered with Bmal1 could release more exosomal miR-22-3p. Moreover, we found that the FABP4 level was elevated in circulation form both LL-induced mouse model and shift nurses. The expression of FABP4 and miR-22-3p in nurse samples exhibited a positive correlation, implying that the upregulated miR-22-3p induced by circadian rhythm disruption at least in part was due to the increased adipocyte-derived exosomes in circulation (Fig. 5, *I*–*K*).

### MiR-22-3p-containing exosomes derived from sh-Bmal1-treated adipocytes promoted IR in skeletal muscle cells

**Figure 6:**
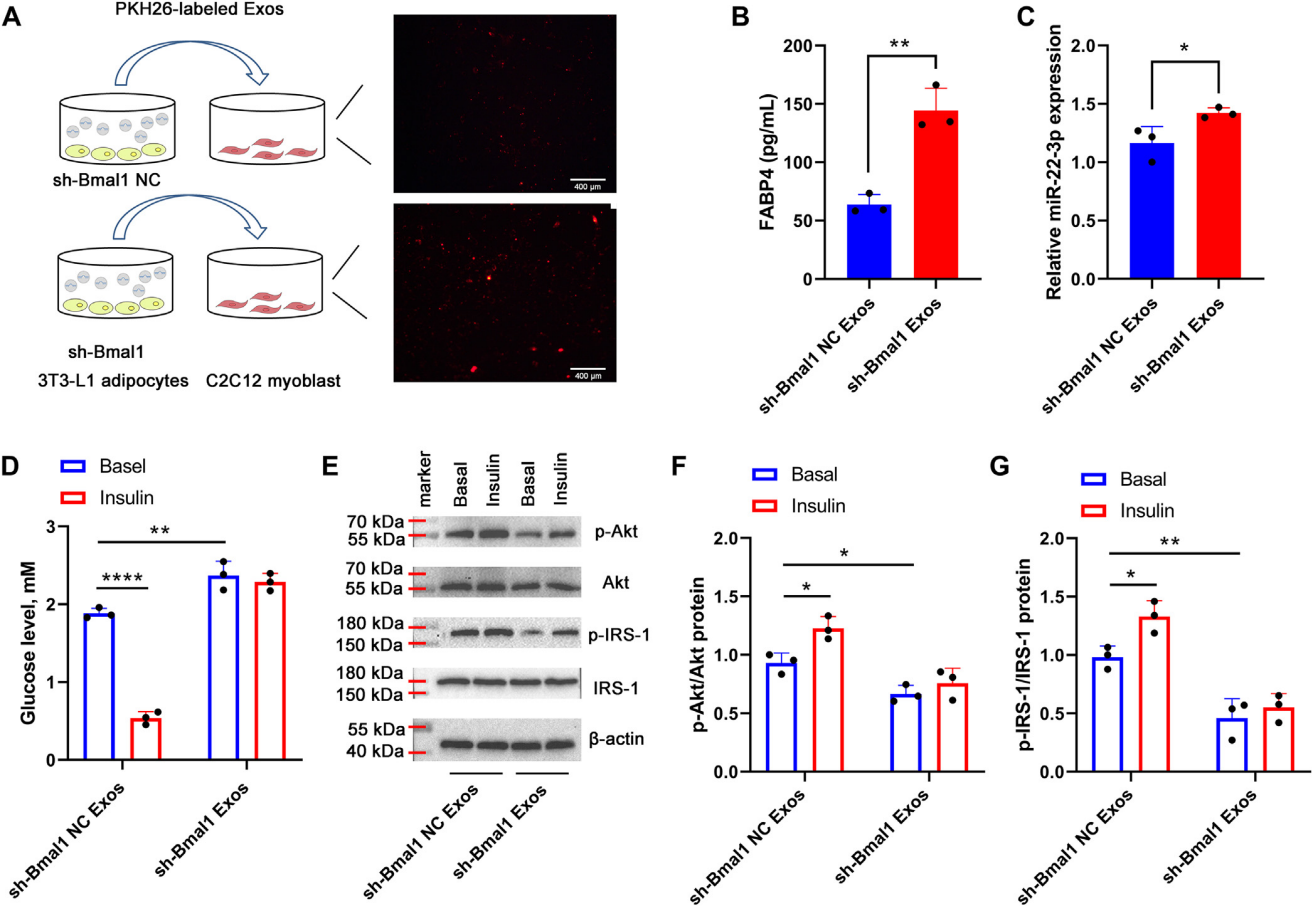
**MiR-22-3p–containing exosomes derived from sh-Bmal1–treated 3T3-L1 adipocytes promoted IR in C2C12 cells.***A*, exosomes isolated from the 3T3-L1adipocytes treated with sh-Bmal1 NC or sh-Bmal1 were labeled with the fluorescent dye PKH26, respectively. These labeled exosomes were then added into the culture medium of C2C12 cells. After 24 h, the presence of red fluorescence staining in C2C12 cells was monitored by a fluorescence microscope (×100). Scale bar represents 400 μm. *B*, the FABP4 level was measured by ELISA. *C*, the expression of miR-22-3p was measured by qPCR. *D*, the glucose levels in the absence or presence of insulin were detected by using a glucose assay kit. *E*, the protein levels of p-Akt, Akt, p-IRS-1, IRS-1 were measured by Western blot assay. The p-Akt/Akt (*F*) and p-IRS-1/IRS-1 (*G*) protein ratio were shown.∗*p* < 0.05; ∗∗*p* < 0.01; ∗∗∗∗*p* < 0.0001.

To determine whether exosomes derived from adipocytes in the context of Bmal1 interference-induced adipogenesis could be taken up by skeletal muscle cells, exosomes isolated from the 3T3-L1adipocytes treated with sh-Bmal1 NC or sh-Bmal1 were labeled with the the fluorescent dye PKH26, respectively. These labeled exosomes were then added into the culture medium of C2C12 cells. After 24 h, the 3T3-L1 adipocytes-derived exosomes could be efficiently taken up by C2C12 cells, as indicated by the presence of red fluorescence staining in C2C12 cells. And the red fluorescence in the sh-Bmal1 Exos group was more than that in the sh-Bmal1 NC Exos group (Fig. 6*A*), validating again that adipocytes interfered with Bmal1 could release more exosomes. Moreover, compared with the sh-Bmal1 NC Exos group, the levels of FABP4 and miR-22-3p expression in the C2C12 cells were both elevated in the sh-Bmal1 Exos group (Fig. 6, *B* and *C*). We further assessed the effects of 3T3-L1 adipocytes-derived exosomal miR-22-3p on the IR of C2C12 cells. As shown in Figure 6*D*, compared with the sh-Bmal1 NC Exos group, the increased miR-22-3p in the sh-Bmal1 Exos group elevated the glucose levels in the supernatant of C2C12 cells, which showed no difference after adding insulin, suggesting the IR occurence. In addition, compared with the sh-Bmal1 NC Exos group, the protein levels of p-Akt and p-IRS-1 in C2C12 cells were significantly decreased in the sh-Bmal1 Exos group with and without insulin treatment (Fig. 6, *E*–*G*), indicating that Bmal1 interference induced more exosomal miR-22-3p releasing form 3T3-L1 adipocytes, which was further absorbed by C2C12 cells to promote IR occurence by suppressing the activation of Akt signaling.

### MiR-22-3p secreted from 3T3-L1 adipocytes in an exosome-dependent manner modulated insulin sensitivity in C2C12 cells

**Figure 7:**
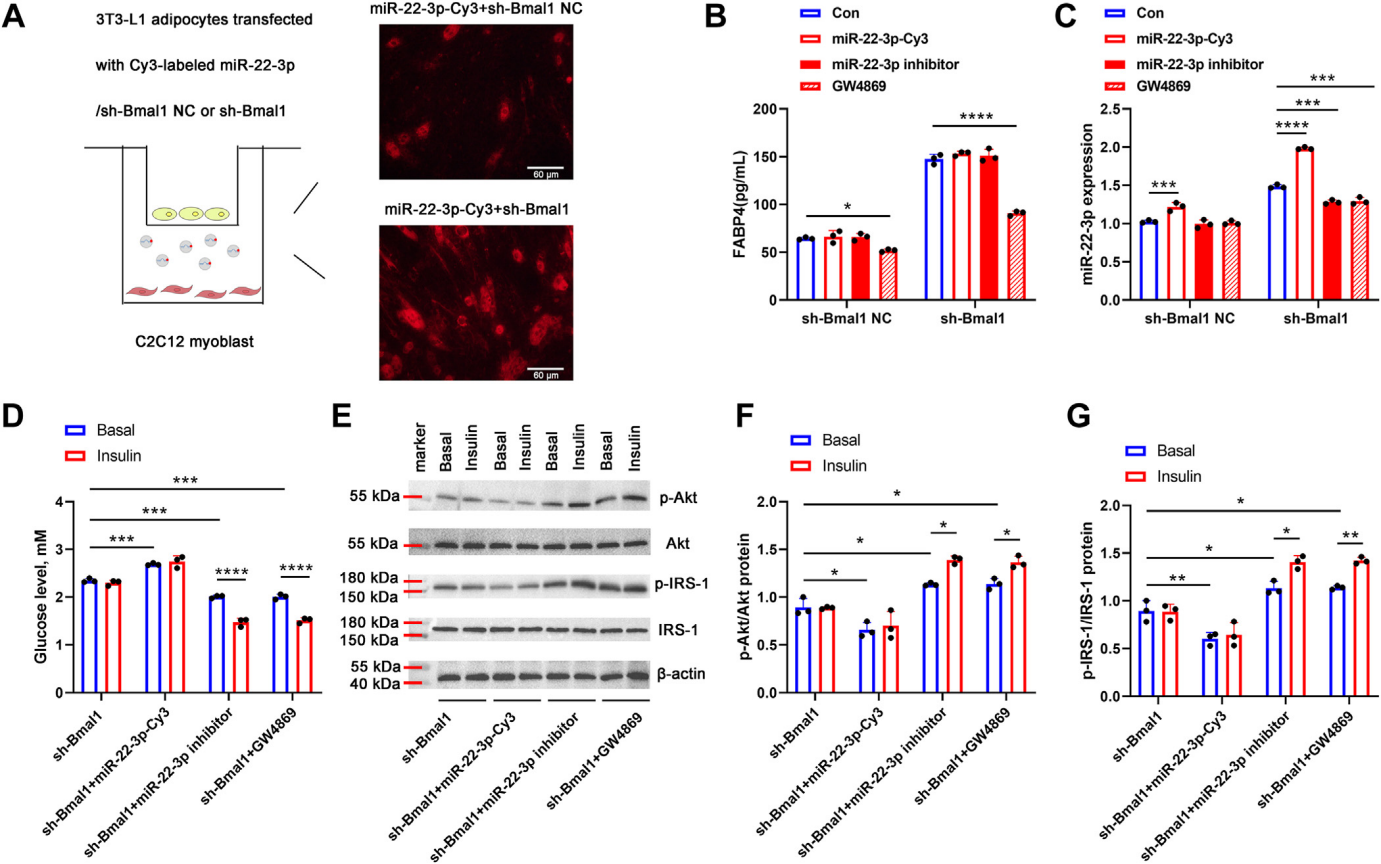
**MiR-22-3p secreted from 3T3-Ll adipocytes in an exosome-dependent manner modulated insulin sensitivity in C2C12 cells.***A*, the 3T3-Ll adipocytes were transfected with Cy3-labeled miR-22-3p mimic, along with sh-Bmal1 NC or sh-Bmal1, and then co-cultured with C2C12 cells. The Cy3 red fluorescence in C2C12 cells was monitored by a fluorescence microscope (×200). Scale bar represents 80 μm. *B*, the FABP4 level in C2C12 cells was measured by ELISA. *C*, the expression of miR-22-3p in C2C12 cells was measured by qPCR. *D*, the glucose levels in the supernatant of C2C12 cells in the absence or presence of insulin were detected by using a glucose assay kit. *E*, the protein levels of p-Akt, Akt, p-IRS-1, IRS-1 were measured by Western blot assay. The p-Akt/Akt (*F*) and p-IRS-1/IRS-1 (*G*) protein ratio were shown. ∗*p* < 0.05; ∗∗*p* < 0.01; ∗∗∗*p* < 0.001; ∗∗∗∗*p* < 0.0001.

To better demonstrate the secretion and uptaken of exosomal miR-22-3p from 3T3-L1 adipocytes to C2C12 cells, the 3T3-L1 adipocytes that transfected with a fluorescent Cy3-labeled miR-22-3p mimic, along with sh-Bmal1 NC or sh-Bmal1, were co-cultured with C2C12 cells. As shown in Figure 7*A*, the appearance of Cy3 red fluorescence in C2C12 cells demonstrated that the miR-22-3p-Cy3 was delivered from the 3T3-L1 adipocytes in the upper well to the recipient C2C12 cells in the lower well. And the red fluorescence in the miR-22-3p-Cy3+sh-Bmal1 group was more than that in the miR-22-3p-Cy3+sh-Bmal1 NC group.

Furthermore, to further confirm the involvement of miR-22-3p in promoting IR occurrence in C2C12 cells, besides the overexpression of miR-22-3p, the 3T3-L1 adipocytes were also transfected with miR-22-3p inhibitor to suppress its level delivered to C2C12 cells. To prove the delivery of miR-22-3p form 3T3-L1 adipocytes to C2C12 cells was due to exosomes, the 3T3-L1 adipocytes were treated with GW4869 to block exosome production. Then these 3T3-L1 adipocytes were co-cultured with the C2C12 cells, respectively, followed by the assessment of miR-22-3p delivery and IR occurrence in the C2C12 cells.

As shown in Figure 7*B*, with or without Bmal1 interference, prior addition of GW4869 to the 3T3-L1 adipocytes decreased FABP4 level in C2C12 cells. The following miR-22-3p detection showed that the miR-22-3p level was both reduced in GW4869 groups with or without Bmal1 interference. Furthermore, miR-22-3p overexpression in the 3T3-L1 adipocytes with or without Bmal1 interference both markedly increased miR-22-3p level in the C2C12 cells; miR-22-3p inhibition in the 3T3-L1 adipocytes with or without Bmal1 interference suppressed the miR-22-3p level in the C2C12 cells, but the difference was significant only when Bmal1 was interfered (Fig. 7*C*). The above results indicated that the delivery of miR-22-3p from 3T3-L1 adipocytes into C2C12 cells depended predominantly on an exosome-dependent manner.

Moreover, as shown in Figure 7, *D*–*G*, miR-22-3p overexpression in 3T3-L1 adipocytes further elevated the glucose levels in the supernatant and down-regulated p-Akt/Akt and p-IRS-1/IRS-1 protein ratio in C2C12 cells co-cultured with sh-Bmal1-tranfected 3T3-L1 adipocytes in the absence or presence of insulin, suggesting the robust role of miR-22-3p modulating insulin sensitivity. However, the decreased miR-22-3p induced by miR-22-3p inhibition or GW4869 treatment in the 3T3-L1 adipocytes suppressed the glucose levels in the supernatant and elevated the p-Akt/Akt and p-IRS-1/IRS-1 protein ratio, which were more significant after adding insulin. The above results demonstrated that in the context of circadian rhythm distribution, miR-22-3p, which was secreted from 3T3-L1 adipocytes in an exosome-dependent manner, was sufficient to suppress insulin sensitivity in C2C12 cells.

## Discussion

With the unveiling of the 2017 Nobel Prize in physiology “Circadian mechanism”, the research in the context of circadian rhythm has become a hot spot in recent years. Light is the most important synchronous stimulator of the circadian clock system, and more than 80% of the world's population is exposed to artificial light pollution (23). Moreover, the coronavirus disease 2019 (COVID-19) pandemic has placed a huge burden on the entire population of every country in the world. Social restrictions, limited daylight exposure, and changes in daily life are strongly associated with sleep and circadian changes (24). It is foreseeable that circadian rhythm disorder will continue to be prevalent in the future, and the associated metabolic abnormalities such as IR should not be ignored. Therefore, it is of great significance to explore the regulatory mechanism of IR in the background of circadian rhythm disorder for the development of effective prevention and therapy targets. Firstly, we established a mouse model of circadian rhythm disruption by prolonged light pollution, which exhibited elevated systemic and skeletal muscle IR. To further clarify the underlying mechanism, we focused on the exosomal miRNAs as their broad and crucial roles in various areas (25, 26, 27, 28, 29, 30). For example, Zheng *et al.* (31) have reported that exosome-mediated miR-155 transfer from smooth muscle cells to endothelial cells destroys tight junctions and the integrity of endothelial barriers, resulting in an elevated endothelial injury and enhanced atherosclerotic progression. Morelli *et al.* (32) have demonstrated that miR-195, a cardiomyocyte-specific miRNA, transferred to fibroblasts in form of exosomal cargo, is crucial in the activation of myofibroblasts. Mills have showed that cancer-derived exosomal miRNAs function as modulators of tumor microenvironment, such as mediating the recruitment and differentiation of cellular components, modulating the vascular permeability and remodeling extracellular matrix (33). The above studies imply that exosome-mediated miRNA transfer is an efficient mode of cell-to-cell communication, in which miRNAs act as paracrine molecules, exerting their regulatory effects in recipient cells. However, there are few studies on exosomal miRNAs in the context of circadian rhythm disruption. In the present study, the dysregulated exosomal miRNAs (miR-22-3p, miR-99a-3p, miR-223-3p, miR-376a-3p, miR-2137 and miR-425-5p) in the plasma exosomes from mouse model of circadian rhythm disruption, which are associated with IR, were screened out through miRNA profile, suggesting that they may deeply participate in regulating IR in the context of circadian rhythm disruption.

Recent studies of exosomes secreted by multiple cells have expanded our understanding of cell communication (11, 34). As an endocrine organ, adipose tissue could release exosomes to communicate with peripheral cells and distant organs, thus regulating metabolic homeostasis. The dialogue between adipose tissue and skeletal muscle has attracted great interest because this process, particularly in obesity, substantially drives the development of muscle IR (35). Considering the dysregulated exosomal miRNAs in circulation, we speculated whether they were involved in the visceral adipose tissues and skeletal muscle tissues. In the present study, qPCR data showed that among these miRNAs, miR-22-3p was significantly upregulated in plasma exosomes, skeletal muscle tissues, and visceral adipose tissues from the mouse model of circadian rhythm disruption, respectively, implying that exosomal miR-22-3p in circulation may be a button molecule that connects the dialogue between adipose tissues and skeletal muscle tissues.

Studies have demonstrated that adipose tissue may be the main source of circulating exosome miRNA in obesity (21, 36). Wei *et al.* (37) have reported that a high fat diet-induced obesity could aggravate colitis because the visceral adipose exosomes efficiently circulated into intestine to promote macrophage M1 polarization via transferring miR-155. In consideration of that LL-induced circadian rhythm disruption led to obesity and visceral fat accumulation, which may further accelerate the release of exosome miRNA into circulation, we speculated whether the upregulated exosomal miR-22-3p in circulation was derived from adipocytes. Thus, to explore the ability of adipocytes releasing exosomal miR-22-3p in the context of circadian rhythm disruption, we established a cell model of circadian rhythm disruption by using Bmal1 interference. As a clock gene, the interference of Bmal1 has been revealed to increase adipogenesis and adipocyte hypertrophy via transcriptional control of Wnt pathway, providing a molecular link between circadian disruption and obesity (22). Consistent with the study, we showed that inhibition of Bmal1 induced the accumulation of lipid in adipocytes, along with the elevated miR-22-3p expression in the supernatants. Furthermore, miR-22-3p and FABP4 (38) in the exosomes from the sh-Bmal1 group exhibited upregulated expression, indicating Bmal1 inhibition-induced adipogenesis could contribute to the release of exosomal miR-22-3p in 3T3-L1 adipocytes. Moreover, the elevated FABP4 level in the plasma form both LL-induced mouse model and shift nurses, as well as the positive correlation between FABP4 and miR-22-3p in the plasma from nurse samples demonstrated that the upregulated miR-22-3p in circulation at least in part was due to the increased adipocyte-derived exosomes in the context of circadian disruption.

Furthermore, the roles of miRNAs in modulating IR has attracted much attention in recent years. A variety of miRNAs exhibited dysregulated expression when IR develops in skeletal muscle, such as upregulated miR-106b, miR-29a and Let-7 or downregulated miR-133a, miR-149 and miR-1. These miRNAs participate in the occurrence and development of skeletal muscle IR through multiple ways including modulating glucose uptake and insulin signaling, reminding us that the upregulated miR-22-3p in skeletal muscle tissues may participate in the regulation of IR. Thus, we further verified the effect of miR-22-3p on IR in skeletal muscle cells. The date demonstrated that miR-22-3p overexpression promoted the occurance of IR by decreasing the protein levels of p-IRS-1 and p-Akt (key proteins in insulin-dependent glucose uptake (39, 40)) with or without insulin, leading to low glucose uptake and high glucose levels in the supernatant of C2C12 cells. Meanwhile, miR-22-3p inhibition suppressed TNF-α-induced IR by increasing the protein levels of p-IRS-1 and p-Akt, leading to high glucose uptake and low glucose levels in the supernatant of C2C12 cells. These data brought us new question that could adipocytes influence IR in skeletal muscle cells by delivering miR-22-3p?

Ying *et al.* (41) have reported that adipose tissue macrophage-derived exosomes in obese mice can induce IR in target cells, including skeletal muscle, through exosomal miR-155. Yu *et al.* (42) have shown that miR-27a released from adipocytes of obese mice induced IR in C2C12 skeletal muscle cells. These studies indicate that adipose tissue-derived exosomal miRNAs are an important mechanism for regulating skeletal muscle IR. Therefore, whether exosomal miR-22-3p derived from adipocytes could act on skeletal muscle and participate in the regulation of IR deserves to be further clarified. In the present study, we showed that exosomes from 3T3-L1 adipocytes (treated with or without Bmal1 interference) could be taken up by C2C12 cells, but more exosomes were absorbed when Bmal1 was interference, verified by the elevated levels of FABP4 and miR-22-3p expression in C2C12 cells. Moreover, the elevated glucose levels in the supernatant of C2C12 cells even though adding insulin were shown in the C2C12 cells treated with sh-Bmal1 Exos, confirming that Bmal1 interference induced more exosomes releasing form 3T3-L1 adipocytes, which was further absorbed by C2C12 cells to promote IR occurrence. Moreover, when Bmal1 was interfered, prior transfection of miR-22-3p mimic in 3T3-L1 adipocytes further elevated the miR-22-3p level in C2C12 cells to enhance IR; prior transfection of miR-22-3p inhibitor or prior addition of GW4869 in 3T3-L1 adipocytes both suppressed the delivery of miR-22-3p from 3T3-L1 adipocytes into C2C12 cells, which further improved insulin sensitivity in C2C12 cells, confirming the robust role of exosomal miR-22-3p derived from adipocytes on IR in skeletal muscle cells in the context of circadian rhythm distribution.

Thirty percent of the workforce in Asia works in shifts, and nurses are the largest group (43). Shift work disrupts circadian rhythm and increases the risk of metabolic disorders (44). Studies have shown that circadian rhythm disruption affects metabolic homeostasis much more in women than in men (45). Female shift nurses have developed abnormal lipid metabolism even if they do not meet overweight/obesity (46). And compared with non-shift nurses, shift nurses showed significantly higher pro-inflammatory factor levels, which play a crucial role in the progression of IR (47, 48). Lee have identified a dose–response relationship between the number of night work years and impaired fasting glucose in non-obese shift workers (49). Consistent with those studies, we found that the CPD (quantitatively assess circadian clock homeostasis) and the IR-relative factors, including VFA, IL-6, FBG, and HOMA-IR, were all significantly increased in the SW group, indicating that shift work affected the stability of circadian clock, and could increase visceral fat and decrease insulin sensitivity before being overweight/obese. Furthermore, miR-22-3p expression and FABP4 level were significantly elevated in the SW group. Considering the previous data that exosomal miR-22-3p derived from adipocytes was deeply involved in the regulation of IR, we speculated that the elevated miR-22-3p expression in circulation might contribute to the decreased insulin sensitivity. We further showed that the expression of miR-22-3p positively correlated with the IR-relative factors, confirming the robust role of miR-22-3p in circulation in the context of circadian rhythm disruption. However, the association of miR-22-3p in circulation with IR in skeletal muscle needs further exploration.

In conclusion, we proposed that exosomal miR-22-3p in circulation may be involved in the regulation of skeletal muscle IR based on the animal model of rhythm disturbance; then combined with the cell model of rhythm disorder, it was clarified that the exosomal miR-22-3p drived from adipocytes acted on skeletal muscle and promoted the occurrence of IR; finally, through the clinical samples of rhythm disorder, the important correlation between exosomal miR-22-3p and IR was confirmed. Considering the advantage for cell communication of exosomes, the present data suggest that exosomal miR-22-3p in circulation may act as potential biomarker and therapeutic target for skeletal muscle IR, contributing to the prevention of T2DM in the context of rhythm disturbance.

### Limitations of the study

Because the experimental background of this paper is relatively complex, which is to explore the regulatory mechanism of skeletal muscle IR under the context of rhythm disturbance, thus the content of this paper involves rhythm disturbance, lipid production, skeletal muscle IR, exosomal miRNA and so on. Due to the many factors involved, it is difficult to unify the modeling methods. For example, LL was used to establish the mouse model of circadian rhythm disruption, but the cell model cannot apply LL induction; We established the cell model of circadian rhythm disruption by using Bmal1 interference; And in clinical experiments, we chose a relatively large group of rhythm disturbance - shift nurses. Thus, the paper involves three modeling methods (animal, cell, clinical experiment). All of these lead to some limitations in this study.

In the study, the biggest limitation is that no *in vivo* experiments have verified that the adipose-derived exosomal miR-22-3p can act on skeletal muscle and induce IR in skeletal muscle in the context of rhythm disturbance, and it would be of interest to further explore whether the effects of circadian rhythm disruption induced by constant light *in vivo* are consistent with that in the *in vitro* cell model of circadian rhythm disruption induced by Bmal1 inhibition in the future. Besides, it is worth exploring the target genes of miR-22-3p to further elucidating molecular mechanisms.

Nevertheless, we could still propose at a macro level that adipocyte-derived exosomal miR-22-3p may contribute to the production of IR in skeletal muscle cells in the context of rhythm disturbance. We presented some evidence at the animal, cellular and clinical levels, hoping to provide some ideas and basis for elucidating the mechanism of skeletal muscle IR in the context of rhythm disturbance.

## Experimental procedures

### Animal experiments

#### Animal model

C57BL/6J mice (male; aged 6–8 weeks) were purchased from Hunan SJA laboratory animal CO, LTD, and divided into two groups, normal illumination (LD) group and continuous illumination (LL) group, randomly. The mice in LD group were housed on a 12-h light-dark cycle (8:00 AM–8:00 PM is the light time), while the mice in LL group were housed under continuous 24-h light (light intensity, 300 lux) (50, 51). 8:00 AM is marked as zeitgeber time (ZT 0). Meanwhile, all the mice were free access to food and water in a animal room (humidity, 50 ± 10%; temperature, 22 °C ± 3 deg.). The CLAMS (Coulumbus Instruments) assay was performed at 10th week. The body weight of each mouse was monitored weekly until week 12 (n = 14). At the 12th week, the mice were anesthetized and the volume of total body fat, subcutaneous fat, and visceral fat were detected by micro-CT (SkyScan 1276). The animal study was approved by the Animal Care and Use Committee of the First Affiliated Hospital of Zhengzhou University (2020-KY-002).

#### CLAMS assessment

The CLAMS was used to observe the food intake and activity patterns of mice (n = 6) and evaluate their energy metabolism, including oxygen consumption (VO_2_), carbon dioxide production (VCO_2_), and respiratory exchange rate (RER), etc. The average RER was calculated as the ratio of VCO_2_:VO_2_ over 48 h.

#### Insulin tolerance test

After 12 weeks of different light treatments, the mice in each group (n = 7) were submitted to 12 h of fasting and administered insulin (0.6 U/kg body weight) by intraperitoneal injection. Then, blood glucose levels were detected at 0, 15, 30, 60, 90, and 120 min by using glucometer (Roche) according to the manufacturer's protocol, and the curve of blood glucose was plotted. AUC was further calculated.

#### Glucose tolerance test

After 12 weeks of different light treatments, the mice in each group (n = 8) were submitted to 12 h of fasting and administered D-glucose (2 g/kg body weight) by intraperitoneal injection. Then, blood glucose levels were measured at 0, 15, 30, 60, 90, and 120 min by using glucometer, and the curve of blood glucose was plotted. AUC was further calculated.

#### Acquisition of serum

For extraction of serum samples, blood was taken immediately after sacrifice, and 1 h at room temperature later, the serum was collected after centrifugation at 3000 rpm for 10 min at 4 °C.

#### Determination of FABP4 level

The FABP4 level in serum was measured using a ELISA kit (R&D Systems) according to the corresponding manufacturer’s instructions, respectively. Protein lysates were measured with a BCA protein assay kit (Beijing Dingguo Changsheng Biotechnology CO, LTD). OD value of each well was measured at 450 nm wavelength. Standard curve was drawn, and the concentration of FABP4 was calculated.

#### Western blot assay

After 12 weeks of different light treatments, the mice in each group (n = 3) were euthanized by cervical dislocation, and skeletal muscle (gastrocnemius muscle) tissues were isolated. The total protein of skeletal muscle tissues were extracted by using RIPA buffer (Beyotime Institute of Biotechnology). Protein quantification was performed using the BCA kit according to the instruction. The protein (15–30 μg) were separated by SDS-PAGE and then transferred onto nitrocellulose membranes. After blocking with 5% nonfat milk for 2 h, the membranes were incubated with target primary antibodies (including anti-IRS-1, anti-p-IRS-1, anti-Akt, and anti-p-Akt) at 4 °C overnight. Then the membranes were incubated with corresponding secondary antibody at room temperature for 1 h. The protein bands were exposed by Bio-Rad exposure instrument using an enhanced chemiluminescence detection kit (Solarbio).

#### Plasma exosome extraction

After 12 weeks of different light treatments, the plasma exosomes from the mice in each group (n = 4) were extracted by using ExoQuick ULTRA EV Isolation Kit for Serum and Plasma (Cat # EQULTRA-20A-1, SBI) according to the protocol. Briefly, ExoQuick ULTRA was added to 250 μl plasma and incubated for 30 min at 4 °C and then centrifuged (3000*g*, 10 min) to remove the supernatant. The exosome-containing pellet was resuspended and added to pre-washed ExoQuick ULTRA columns, followed by centrifugation (1000*g*, 30 s) and exosome collection.

#### Identification of exosomes

The total protein of exosomes were extracted by using RIPA buffer. Protein quantification was performed using the Micro BCA Protein Assay Kit (Cat.#23235, Thermo Fisher Scientific) according to the instruction. Western blot analysis was performed to determine the expression of CD9, CD63, Alix and Calnexin. For the morphology observation, the exosomes were fixed with 2% paraformaldehyde, and then loaded on Formvar and carbon-coated copper grids. Then the grids were placed on 50 μl 1% glutaraldehyde droplets for 5 min, after rinsing with ddH_2_O, the grids were placed on 50 μl uranium dioxyoxalate droplets. After 5 min, the grids were placed on 50 μl methyl cellulose droplets for 10 min. After drying for 5 min, the grids were viewed and photographed using a transmission electron microscope. Furthermore, the particle size distribution of exosomes was detected by nanoparticle tracking analysis (NTA) using ZetaView PMX 110 (Particle Metrix) according to the instruments.

#### Exosomal miRNA sequencing

Exosomal miRNA sequencing and bicluster analysis of the differentially expressed miRNAs were performed by Personalbio.

#### qPCR assay

The RNAs from plasma exosomes were extracted by ExoQuick Exosome Isolation and RNA Purification Kit (for Serum & Plasma) (System Biosciences). Meanwhile, after 12 weeks of different light treatments, the skeletal muscle (gastrocnemius muscle) tissues and epididymal visceral adipose tissues from mice in each group (n = 3) were isolated, respectively. Then the total RNAs were extracted using the Trizol reagent (Qiagen). Complementary DNA was reverse transcribed using HiScript II Q RT SuperMix for qPCR (Vazyme). The tissular or exosomal miRNA expression was measured using ChamQTM Universal SYBR qPCR Master Mix (Vazyme) following the manufacturer's instructions. Data were normalized to levels of U6, and the relative expression of target miRNA was analyzed by 2^−△△CT^ method. The primer sequences are presented in Table S1.

#### Statistical analysis

SPSS 22.0 software and GraphPad Prism 8.0 software were used for statistical analysis. Data are expressed as mean ± SD. The statistical significance of the differences between various groups was measured by either independent-samples *t* test with Shapiro-Wilk test and Levene test (two groups) or two-way ANOVA with Tukey's multiple comparisons test (more than two groups). The differential miRNA was mainly screened by Fisher's Exact test. A value of *p* < 0.05 was considered statistically significant.

### Clinical experiments

#### Human samples

Twelve shift nurses (shift working group, SW group) in the department of blood transplantation and 12 non-shift nurses (non-shift working group, NSW group) in the department of physical examination were collected from the First Affiliated Hospital of Zhengzhou University from June to September 2020. In this study, shift nurses regularly rotated different shifts: day shift (08:00∼16:00), evening shift (16:00∼24:00), night shift (24:00∼08:00) to achieve nursing work continuity. Inclusion criteria: (1) SW group: healthy women aged 18 to 30 years, shift duration ≥1 year, night shift duration ≥5 times per month, sleep duration of night shift <6 h, 18.5 kg/m^2^ ≤ body mass index ≤ 24 kg/m^2^; (2) Non-shift working group: healthy women aged 18 to 30 years, sleep time ≥7 h per day, do not engage in shift work, 18.5 kg/m^2^ ≤ body mass index ≤ 24 kg/m^2^, sleep and diet regular. Exclusion criteria: Complicated with chronic diseases (diabetes, hypertension, hyperlipidemia, coronary heart disease, etc.); Stress diseases such as inflammation or infection in the past 1 month; Smoking; Habitual excessive drinking; Insomnia; Received general anesthesia in the past 1 month; Drug use history in the past 3 months; Pregnancy or lactation. Informed consents were obtained from all participants, and this study was approved by the Clinical Ethics Committee of the First Affiliated Hospital of Zhengzhou University (2019-KY-318).

#### Body mass index and visceral fat area

Body composition analyzer (Inbody770, Inbody Co, LTD) was used for detection. Human subjects were measured on an empty stomach, before examination, and when calm breathing. Measurement indicators include height, weight, body mass index, VFA, etc.

#### Measurement of miR-22-3p level

The total RNA in serum was extracted by Trizol method and reverse transcribed into complementary DNA using HiScript II Q RT SuperMix for qPCR (R223-01, Vazyme). The miR-22-3p expression was measured using ChamQTM Universal SYBR qPCR Master Mix (Vazyme) following the manufacturer's instructions. U6 was used as the internal reference for standardization, and the relative miR-22-3p expression was analyzed by 2^−△△CT^ method. The primer sequences are presented in Table S1.

#### Analysis of CPD

CPD was used to quantify social jet lag and quantitatively assess circadian clock homeostasis. The midsleep was calculated based on the time of falling asleep and waking up in the 1-week sleep questionnaire. NSW group was calculated according to the midsleep on weekday and rest day, and SW group was calculated according to the midsleep on weekday and rest day after night shift. Calculation formula: |CPD_i_| = (X_i_^2^ + Y_i_^2^)^0.5^. CPD_i_ = CPD on day i; X_i_ = Distance of midsleep on day i to chronotype (midsleep on rest day or rest day after night shift); Y_i_ = Distance of midsleep on day i to previous day i − 1 (52).

#### Evaluation of biochemical indexes

Fasting venous blood was collected between 7 and 8 o 'clock in the morning on the day of the end of night shift in the SW group, and blood was collected at the same time in the NSW group. FBG was detected by glucose dehydrogenase method, and the kit was purchased from Roche of Germany; Fasting insulin was detected by chemiluminescence microparticle immunoassay with a kit purchased from Abbott of USA; Homeostasis model assessment of IR (HOMA-IR) = FBG (mmol/l) × fasting insulin (μIU/ml)/22.5 (53); Serum interleukin-6 (IL-6) and FABP4 levels were measured by ELISA kits, purchased from R&D Systems of USA. The sample concentrations were then calculated based on absorption density determined at 450 nm.

#### Statistical analysis

SPSS 22.0 software and GraphPad Prism 8.0 software were used for statistical analysis. Data are expressed as mean ± SD. The statistical significance of the differences between two groups was measured by independent-samples *t* test with Shapiro-Wilk test and Levene test. Spearman correlation analysis was used to analyze the correlations between miR-22-3p expression and IR-relative factor levels. A value of *p* < 0.05 was considered statistically significant.

### Cell experiments

#### Cell culture and differentiation

Mouse pre-adipocyte fibroblast 3T3-Ll and mouse myoblast C2C12 cell lines were purchased from Chinese Academy of Sciences, which were both cultured in Dulbecco’s modified Eagle’s medium (DMEM) containing 10% fetal bovine serum (FBS, Gibco) in an atmosphere of 5% CO_2_ at 37 °C. Cell passage was carried out when cell confluence reached 80% to 90%.

To generate adipocytes, the 3T3-Ll cells were introduced to differentiate after 48 h of contact inhibition. 3T3-Ll cells were differentiated in DMEM medium containing 10% FBS, 1 μM dexamethasone, 0.5 mM 3-isobutyl-1-methylxanthine and 10 μg/ml insulin for 7 to 8 days. When circular lipid droplets were observed under the microscope, DMEM medium (containing 10% FBS and 10 μg/ml insulin) was used for 48 h of differentiation. Then the differentiation medium was abandoned, and the culture medium was changed to DMEM medium containing 10% FBS, and the culture medium was changed every 48 h for 48 to 72 h. The formation of lipid droplets in 3T3-Ll cells was observed under a microscope (Olympus).

The C2C12 cells (grown to 90% confluence) were differentiated by incubation with DMEM containing 2% horse serum (Gibco) for 4 to 7 days. The myotube formation was observed under a microscope.

#### Cell model of IR

The establishment of IR cell model was performed as previously described (54). Briefly, the differentiated C2C12 cells were pretreated with high-glucose DMEM containing 0.5% FBS for 3 h. The obtained cells were further treated with high-glucose DMEM containing 10% FBS supplemented with 10 ng/ml TNF-α for 1 day. The glucose level was measured by a glucose (GO) assay kit (Sigma-Aldrich).

#### Cell transfection

Adenovirus vectors encoding short hairpin (sh)-Bmal1 was designed and constructed by Shanghai Genechem Co, LTD. Briefly, the sequences of sh-Bmal1 were synthesized and then inserted into adenovirus shuttle vector GV119 (including an EGFP) through the two restriction sites Age I and EcoR I. After verifying the plasmids by sequencing, they were transfected into HEK293A cells, together with helper plasmids, by Lipofectamine 2000 (ThermoFisher Scientific). After collection, purification and titer determination, the linearized adenoviral DNA was used to transfect the differentiated 3T3-Ll cells. No load adenovirus was used as negative control (sh-Bmal1 NC). After 72 h, the fluorescence intensity was observed and photographed to assess the infection efficiency. Western blot was performed to determine the ability of interfering Bmal1 expression.

MiR-22-3p mimic (Cy3 labeled or not), miR-22-3p inhibitor, and their negative controls (Ribo Bio Technology Co, Ltd) were transfected into C2C12 cells with the Lipofectamine 2000, respectively. After 24 h, the transfection efficiencies were validated by either qPCR or Western blot analysis. The oligonucleotide sequences are presented in Table S2.

#### Oil red O staining

Oil red-O staining, which stains for neutral lipids, was used to assess lipid accumulation as a result of adipogenic differentiation. The oil red O solution was prepared with the stock solution of oil red O (Solarbio) and distilled water in a ratio of 3:2, and filtered before use. The 3T3-Ll cells were washed with PBS, and fixed in 4% formaldehyde for 30 min, and then stained with oil red O solution at 37 °C for 1 h. Excess stain was washed with distilled water, and the cells were photographed using a microscope.

#### Determination of triglyceride level

Intracellular TG content was determined by glycerol 3-phosphate oxidase method using a TG determination kit as per the manufacturer’s instructions (Cayman Chemical Company).

#### Extraction and identification of exosomes in the supernatant

Exosomes from the supernatant were extracted and purified by using an exoEasy Maxi Kit (Qiagen) according to the manufacturer’s instructions. The characterization of exosomes was identified by measuring expression of exosome-associated protein markers CD9, CD63, Alix and Calnexin by Western blot.

#### *In vitro* exosome treatment

To monitor exosome trafficking, 500 μl exosomes from each group were labeled with PKH26 fluorescent dye (Umibio). For *in vitro* treatment, PKH26-labeled exosomes from each group were added to 3 × 10^5^ C2C12 cells, respectively. After 24 h, the absorption of exosomes by C2C12 cells was observed and photographed by fluorescence microscopy (Olympus).

#### Co-culture assay

To better demonstrate the secretion of exosomal miR-22-3p, miR-22-3p mimic were labeled with Cy3 fluorescent dye, and then the differentiated 3T3-Ll adipocytes were transfected with Cy3-miR-22-3p mimic for 24 h, followed by transfection of adenovirus-sh-Bmal1 or adenovirus-sh-Bmal1 NC for 48 h. The transfected 3T3-Ll adipocytes were co-cultured with C2C12 cells at a ratio of 1:1 using a transwell plate (0.4 μm polycarbonate filter, Corning), with C2C12 cells placed in the lower chamber and 3T3-Ll adipocytes in the upper chamber. After 48 h, the appearance of Cy3 red fluorescence on C2C12 cells was photographed.

To inhibit exosome secretion, 3T3-Ll adipocytes were pretreated with exosome secretion inhibitor GW4869 (an inhibitor of neutral sphingomyelinase, 10 μM) for 24 h, followed by transfection of adenovirus-sh-Bmal1 or adenovirus-sh-Bmal1 NC for 48 h. Then these 3T3-Ll adipocytes were co-cultured with C2C12 cells for another 48 h.

To clarify that miR-22-3p secreted from exosomes of 3T3-Ll adipocytes is the sole source of miR-22-3p upregulation in C2C12 cells, 3T3-Ll adipocytes were pretransfected with miR-22-3p inhibitor for 24 h, followed by transfection of adenovirus-sh-Bmal1 or adenovirus-sh-Bmal1 NC for 48 h. Then these 3T3-Ll adipocytes were co-cultured with C2C12 cells for another 48 h.

#### qPCR assay

Total RNAs were extracted from 3T3-Ll adipocytes or C2C12 cells using Trizol method. The RNAs of 3T3-Ll adipocytes-derived exosomes were extracted by ExoQuick Exosome Isolation and RNA Purification Kit (for Serum & Plasma). The cellular mRNA and miR-22-3p or exosomal miR-22-3p expression were measured as described above. Data were normalized to levels of GAPDH (mRNA) or U6 (miR-22-3p). The primer sequences are presented in Table S1.

#### Determination of FABP4 level

The levels of FABP4 in the exosomes, and lysates of C2C12 cells were measured using ELISA (Abcam) as mentioned above.

#### Measurement of glucose level

The glucose levels in the supernatant in the absence or presence of insulin (100 nM, 0.5 h) were measured using a glucose (GO) assay kit according to the manufacturer’s procedure (Sigma-Aldrich). Absorbance at 540 nm was read by using a conventional plate reader, which was proportional to the concentration of glucose.

#### Western blot assay

Total protein of C2C12 cells were extracted by RIPA buffer and quantified by a BCA kit. The expression of IRS-1, p-IRS-1, Akt, and p-Akt were then determined by SDS-PAGE as described above.

#### Statistical analysis

SPSS 22.0 software and GraphPad Prism 8.0 software were used for statistical analysis. Data are expressed as mean ± SD. The statistical significance of the differences between various groups was measured by either the independent-samples *t* test with Shapiro-Wilk test and Levene test (two groups) or one-way ANOVA, two-way ANOVA with Tukey's multiple comparisons test (more than two groups). A value of *p* < 0.05 was considered statistically significant.

## Data availability

All data are available from the authors upon request.

## Supporting information

This article contains supporting information.

## Conflict of interest

The authors declare that they have no conflicts of interest with the contents of this article.
