# Supplementary material for: Adipocyte-derived exosomal miR-22-3p modulated by circadian rhythm disruption regulates insulin sensitivity in skeletal muscle cells
Source: J Biol Chem. 2023 Nov 18;299(12):105476. doi: 10.1016/j.jbc.2023.105476 (PMC10750178; doi:10.1016/j.jbc.2023.105476)
Supplement: Tables S1–S3 [file mmc1.docx]

**Supplementary Table 1 Primer sequences**

| **Gene** | **Primer Sequence（5’→3’）** |
| --- | --- |
| miR-22-3p-R | GTCGTATCCATGGCAGGGTCCGAGGTATTCGCCATGGATACGACACAGTT |
| miR-22-3p-F | AAGCTGCCAGTTGAAG |
| miR-99a-3p-R | GTCGTATCCATGGCAGGGTCCGAGGTATTCGCCATGGATACGACAGACCC |
| miR-99a-3p-F | CAAGCTCGTTTCTATG |
| miR-223-3p-R | GTCGTATCCATGGCAGGGTCCGAGGTATTCGCCATGGATACGACTGGGGT |
| miR-223-3p-F | TGTCAGTTTGTCAAAT |
| miR-376a-3p-R | GTCGTATCCATGGCAGGGTCCGAGGTATTCGCCATGGATACGACACGTGG |
| miR-376a-3p-F | ATCGTAGAGGAAAATC |
| miR-425-5p-R | GTCGTATCCATGGCAGGGTCCGAGGTATTCGCCATGGATACGACTCAACG |
| miR-425-5p-F | AATGACACGATCACTC |
| miR-2137-R | GTCGTATCCATGGCAGGGTCCGAGGTATTCGCCATGGATACGACCTCCCT |
| miR-2137-F | GCCGGCGGGAGCCCCA |
| U6-R | TTCACGAATTTGCGTGTCAT |
| U6-F  Fatp1-R  Fatp1-F  Cpt1b-R  Cpt1b-F  Fasn-R  Fasn-F  Scd1-R  Scd1-F  GAPDH-R  GAPDH-F | TCGCTTCGGCAGCACATA  GATGCACGGGATCGTGTCT  CGCTTTCTGCGTATCGTCTG  CAGGAGTTGATTCCAGACAGGTA  GCACACCAGGCAGTAGCTTT  TGGGTAATCCATAGAGCCCAG  GGAGGTGGTGATAGCCGGTAT  CGGGATTGAATGTTCTTGTCGT  TTCTTGCGATACACTCTGGTGC  CCGTATTCATTGTCATACCAGG  ACTCTTCCACCTTCGATGC |

**Supplementary Table 2 Oligonucleotide sequences**

| **Oligonucleotide** | **Sequence（5’→3’）** |
| --- | --- |
| Con-mimic-sense  Con-mimic-antisense  miR-22-3p mimic-sense  miR-22-3p mimic-antisense  inhibitor-NC  miR-22-3p inhibitor | UUUGUACUACACAAAAGUACUG  CAGUACUUUUGUGUAGUACAAA  AAGCUGCCAGUUGAAGAACUGU  AGUUCUUCAACUGGCAGCUUUU  CAGUACUUUUGUGUAGUACAAA  ACAGUUCUUCAACUGGCAGCUUUU |

**Supplementary Table 3 Clinical parameters and biochemical indexes in serum**

|  | NSW | SW |
| --- | --- | --- |
| Height (cm) | 165.08±0.72 | 165.50±0.87 |
| Weight (kg) | 54.46 ±1.02 | 60.15±2.24* |
| BMI (kg/m^2^) | 19.98±0.35 | 21.94±0.75* |
| VFA(cm^2^) | 65.74±3.32 | 92.83±8.51* |
| CPD | 2.08±0.08 | 3.87±0.09*** |
| FBG | 4.73±0.93 | 5.18±0.13* |
| HOMA-IR  IL-6 | 1.46±0.13  48.43±6.66 | 1.85±0.11*  74.46±6.84* |

**P*<0.05, ****P*<0.001 vs NSW group
